# Genome-Wide Profiling of Polyadenylation Events in Maize Using High-Throughput Transcriptomic Sequences

**DOI:** 10.1534/g3.119.400196

**Published:** 2019-06-25

**Authors:** Zehra Jafar, Salma Tariq, Irfan Sadiq, Tayyab Nawaz, Malik Nadeem Akhtar

**Affiliations:** * Computational Biology and Bioinformatics Laboratory, Department of Biosciences, COMSATS University Islamabad, Islamabad 45550, Pakistan; † Department of Mathematics, COMSATS University Islamabad, Islamabad 45550, Pakistan

**Keywords:** *Zea mays*, Alternative polyadenylation (APA), lncRNA, non-coding RNA, 3′-UTR annotations

## Abstract

Polyadenylation is an essential post-transcriptional modification of eukaryotic transcripts that plays critical role in transcript stability, localization, transport, and translational efficiency. About 70% genes in plants contain alternative polyadenylation (APA) sites. Despite availability of vast amount of sequencing data, to date, a comprehensive map of the polyadenylation events in maize is not available. Here, 9.48 billion RNA-Seq reads were analyzed to characterize 95,345 Poly(A) Clusters (PAC) in 23,705 (51%) maize genes. Of these, 76% were APA genes. However, most APA genes (55%) expressed a dominant PAC rather than favoring multiple PACs equally. The lincRNA genes with PACs were significantly longer in length than the genes without any PAC and about 48% genes had APA sites. Heterogeneity was observed in 52% of the PACs supporting the imprecise nature of the polyadenylation process. Genomic distribution revealed that the majority of the PACs (78%) were located in the genic regions. Unlike previous studies, large number of PACs were observed in the intergenic (n = 21,264), 5′-UTR (735), CDS (2,542), and the intronic regions (12,841). The CDS and introns with PACs were longer in length than without PACs, whereas intergenic PACs were more often associated with transcripts that lacked annotated 3′-UTRs. Nucleotide composition around PACs demonstrated AT-richness and the common upstream motif was AAUAAA, which is consistent with other plants. According to this study, only 2,830 genes still maintained the use of AAUAAA motif. This large-scale data provides useful insights about the gene expression regulation and could be utilized as evidence to validate the annotation of transcript ends.

Polyadenylation is a crucial cellular process to generate mature 3′ ends of the nascent transcripts. Maturation of 3′ ends begins with the endonucleolytic cleavage of the nascent transcripts followed by the addition of several adenosine residues to the free 3′ end of the transcript (Di Giammartino *et al.* 2011; Tian and Manley 2017; Chen *et al.* 2017). The location on the transcript where cleavage occurs is known as poly(A) cleavage site and the newly added stretch of A-residues is known as the poly(A) tail. The poly(A) tail provides stability to the transcript, promotes the translational efficiency, plays role in the transport of transcript from nucleus to cytoplasm, and determines localization. Polyadenylation is a widespread modification that occurs in almost all-eukaryotic transcripts and many non-coding RNA transcripts that are encoded by RNA polymerase II (Gruber *et al.* 2014; Tian and Manley 2017; Chen *et al.* 2017).

Most genes in eukaryotic species use more than one poly(A) sites, a phenomenon known as the alternative polyadenylation (APA). The configuration of APA sites could lead to transcripts with altered 3′-UTRs (when multiple sites occur in the same last exon) or altered final protein products (when sites occur in different exons or intronic regions) (Wu *et al.* 2011). Genome-wide polyadenylation studies in different species have revealed the widespread nature of APA events. At least two poly(A) sites have been detected in 70% of the mammalian genes (Derti *et al.* 2012) and approximately 50% of the genes in flies (Smibert *et al.* 2012), and about 80% of the genes in *Danio rerio* (Ulitsky *et al.* 2012). In *Chlamydomonas reinhardtii*, 68% of the genes use APA sites (Zhao *et al.* 2014). Similarly, in higher plants, 64% of *Medicago truncatula* genes and 70% of *Arabidopsis thaliana* genes have more than one poly(A) sites (Wu *et al.* 2011, 2014). Studies based on poly(A) tag sequencing (PAT-seq), expressed sequenced tags (ESTs), and massively parallel signature sequencing (MPSS) place the number of *Oryza sativa* genes using APA sites between 48–82% (Shen *et al.* 2008, 2011; Fu *et al.* 2016).

The choice of a particular poly(A) site is triggered by the interaction between *cis*-elements and polyadenylation machinery (also known as *trans*-elements). The *trans*-elements and *cis*-elements of plants are largely known (Hunt *et al.* 2012; Wang *et al.* 2018). *Cis*-elements in plant are less conserved than the ones present in mammals. Near Upstream Element (NUE) is the most conserved *cis*-element usually positioned between 10 and 40 nucleotides (nts) upstream from the poly(A) sites (Loke *et al.* 2005). In terms of sequence similarity and relative strength, NUE is analogous to mammalian polyadenylation signal AAUAAA (Tian and Graber 2012). However, hexamer AAUAAA is known to be present in only 9–15% of the plant poly(A) sites (Loke *et al.* 2005; Li and Du 2014). The Far Upstream Element (FUE) controls the efficiency of polyadenylation sites and is found between 30-100 nts upstream of the poly(A) site. Overall, the FUE elements are considered less conserved (Rothnie 1996). The composition of plant cleavage site revealed the preference of YA (CA or UA) dinucleotide that is located between two U-rich region known as cleavage elements (Loke *et al.* 2005; Li and Du 2014).

Maize (*Zea mays*; corn) is an important cereal crop of great agronomic value that has been considered as model organism for various genomic studies (Strable and Scanlon 2009). Recently, motif distribution and nucleotide composition has been analyzed in 10,491 maize poly(A) sites generated from the NCBI transcripts (Li and Du 2014). The nucleotide composition displayed a characteristic “U-A-U-A-U” rich pattern that is conserved across animals, plants, and microorganisms. The canonical motif AAUAAA is predominant in the NUE region of the poly(A) sites in maize (Li and Du 2014). Similarly, *Wang et al* observed that APA is common and the AAUAAA or its variants are predominant the motifs in the 3′-UTR region of the full-length transcripts assembled from the RNA-Seq reads in maize (Wang *et al.* 2018). However, both these studies were limited in several aspects. First, both studies only tested 3′-UTR poly(A) sites and provide no information about the genomic distribution of “non 3’-UTR” poly(A) sites. Second, none of the studies reported the extent of APA in the maize genome. Considering the importance of APA in gene expression regulation, understanding how the choice of APA is regulated is important. Third, no information has been available about the heterogeneity in the maize genome.

Here, we present a comprehensive catalog of the genome-wide polyadenylation events in maize based on 9.48 billion RNA-Seq reads obtained from the NCBI SRA database. The reads were mapped to maize genome and 95,345 distinct polyadenylation events are identified. The analysis presents the annotation, genome wide distribution, and extent of heterogeneity, single nucleotide profiles, and NUE motif distribution of polyadenylation events in maize. Furthermore, the vast majority of the maize genes has more than one poly(A) sites and could contribute in overall transcriptome and proteome diversity.

## Materials and Methods

### Data collection and determination of polyadenylation sites

The RNA-Seq datasets used to identify polyadenylation events in the B73 variety of maize were acquired from the Sequencing Read Archive (SRA; https://www.ncbi.nlm.nih.gov/sra) database. Details of these datasets (as available in SRA database) are provided in the File S1 and a brief description of each dataset is provided in the File S2. The RNA-Seq data were extracted from the SRA files in the FASTQ format using the *Fastq-dump* software of the *SRA Toolkit* (version 2.8.0) (Leinonen *et al.* 2011). For the paired end data, the additional “–*split-files*” parameter was used to extract the first and second reads separately. *Trimmomatic* software (version 0.36) was used to evaluate the read quality and read lengths (parameters: ILLUMINACLIP:AdapterFile:2:30:10 SLIDINGWINDOW:4:15 MINLEN:25) (Bolger *et al.* 2014).

The reads containing eight or more A-residues at the 3′ end or T-residues at 5′ end (according to the strand specificity) were defined as the polyadenylated reads as described previously (Wang *et al.* 2016b). Briefly, the *FindTail* program (Dong *et al.* 2015) with “–endgap 2” parameter was used to select the polyadenylated reads. This script identifies the perfect poly(A) sequences at first and then it combines the sequences using an user-defined size of non-A bases to form a longer poly(A) tail. For the non-stranded data, reads with at least eight A-residues at the 3′ end or T-residues at the 5′ end were selected. For the strand-specific data, the first reads with at least eight T-residues at the 5′ end or second reads with at least eight A-residues at the 3′ end were selected. The N (Uncalled) residues in the polyadenylated tail were replaced randomly with A, T, C, and G using the *ReplaceN* script. The reads with up to 10% of non-A or non-T residues were finally selected as the polyadenylated reads to account for the possible imperfect homopolymeric tails (Dong *et al.* 2015).

The poly(A) tails in the reads were added post-transcriptionally and should not map to the genome (Zhao *et al.* 2014). To ensure that the detected poly(A) tails were post-transcriptional, the reads from each dataset were mapped to the maize B73 (V4) reference genome (ftp://ftp.gramene.org/pub/gramene/release-55/fasta/zea_mays/dna) using the *Bowtie2* software (Langmead and Salzberg 2012). The *Bowtie2* alignments were performed in the “end-to-end” mode with stringent settings (-D 15 -R 2 -L 22 -i S,1,1.15 -N 1). The “*–un*” parameter of the *Bowtie2* was used to retrieve alignment results of the reads that failed to map the genome in SAM format (Li *et al.* 2009).

In the subsequent step, *TrimPolyA* program (Dong *et al.* 2015) (released on 20-12-2013) was used to trim terminal A- or T-residues from the extracted reads and the trimmed reads that were less than 20 nts in length were discarded. The remaining trimmed reads were mapped with the maize genome using *Bowtie2* in the “end-to-end” mode with stringent settings (-D 15 -R 2 -L 22 -i S,1,1.15 -N 1). Only uniquely mapped reads were retained. The downstream genomic regions of the mapped reads were checked with the *CleanSam.pl* script to filtered out the internally primed reads (Dong *et al.* 2015). The filtered reads from all datasets were pooled together to generate the unique genomic coordinates, which were termed as cleavage sites.

As most of the cleavage sites exist in the form of clusters due to imprecise nature of the cleavage and polyadenylation process (Pauws *et al.* 2001), iterative clustering approach was employed to cluster adjacent cleavage sites that were observed within 24 nts of each other on the same chromosome and strand (Tian *et al.* 2005). Only high quality cleavage sites with ≥3 reads were clustered and rest were discarded. Within each poly(A) site cluster (PAC), the cleavage site having the highest number of mapped reads was considered as the representative cleavage site of the cluster and sum of the mapped reads from all individual cleavage sites was used as the support for the cluster.

### Annotation of PACs and de novo transcript assembly

The respective genic locations of PACs were crucial to distinguish between different types of PACs identified in this study. The latest gene annotations for the B73 variety of the maize were downloaded from the Ensembl (ftp://ftp.ensemblgenomes.org/pub/release-39/plants/gff3/zea_mays). For the genes with multiple transcripts, only the longest transcript was used (Zhao *et al.* 2014; Wang *et al.* 2017). These transcript annotations were used to identify the relative genomic locations of 3′-UTR, 5′-UTR, introns, exons, CDS, and intergenic regions in the maize genome. The intergenic region was defined as the un-annotated region between two genes on the same strand and chromosome. Previous polyadenylation studies (Wu *et al.* 2011; Zhao *et al.* 2014) in plants highlighted possible incompleteness of the 3′-UTR annotations which can lead to the inaccurate classification of genic PAC as intergenic PAC (Wu *et al.* 2015). The 3′-UTR regions were extended using the median of the intergenic PAC distribution to improve annotations (Zhao *et al.* 2014).

To further validate the intergenic PACs, *de novo* transcripts were assembled in the maize genome using 68 strand-specific RNA-Seq samples obtained at different developmental stages from the multiple tissues (SRP029238). *Trimmomatic* software was used to remove the adapters and filter low quality reads (parameters: ILLUMINACLIP:AdapterFile:2:30:10 SLIDINGWINDOW:4:15 MINLEN:25). Only reads that were greater than 25 nts in length were retained further. The quality filtered reads were aligned onto the reference maize genome using HISAT2 (version 2.1.0; flags:–rna-strandness R -q). The HISAT2 alignments in SAM format were converted into BAM format followed by sorting using SAMtools (version 1.3.1). The sorted BAM files were used to identify transcripts from each RNA-Seq sample with StringTie (version 1.3.5; flag:–rf) using Ensembl transcripts as a reference guide. The transcriptome assemblies from all samples were merged together using StringTie “merged” option in a reference-guided manner to generate a master transcriptome (transcripts = 242,307 and genes = 73,965). Of these genes, 12,719 overlapped completely with Ensembl annotations, whereas remaining 61,246 genes either had all novel transcripts (41,095 genes) or at least one novel transcript (20,151 genes). Only genes with at least one novel transcript (n = 61,246 genes) were considered further to validate intergenic PACs. Multiple transcripts from the same gene were merged and the 5′-most and 3′-most transcript termini were used to define the gene start and end coordinates. The intergenic PACs that were located within the assembled *de novo* gene coordinates were considered as the validated PACs.

### Analysis of PAC expression levels in APA genes

The genes with two or more PAC were considered as the APA genes. The relative expression level (RE) of the PACs within each APA gene was measured as the ratio of the number of unique poly(A) reads mapped to a given PAC *vs.* the total number of the mapped poly(A) reads on that gene. Using a gene-centric approach (Zhao *et al.* 2014), any PAC with an RE value greater than an empirically selected threshold of 0.7 was categorized as the “strong” PAC, while all other PACs in that gene were categorized as “weak” PACs. All the PACs in a gene with no “strong” PAC were considered as the “medium” PACs. Similarly, gene with strong PACs was categorized as “strong” gene, while the genes without a strong PAC were classified as the medium gene. The PACs from each gene were ranked in descending order based on their RE values. For PAC having the same rank from all genes, the total supporting reads were estimated and divided by the total reads mapped to all APA genes to calculate the fraction of mapped reads.

### Analysis of sequences Around PACs

The [-300:+100] sequence around each PAC was extracted from the genome. Single nucleotide composition in the extracted sequences were analyzed using the *SignalSleuth2* software with parameters k = 1 and gap = 0 (Zhao *et al.* 2014). The deviations in the single nucleotide composition of PACs found in different types of genomic regions (CDS, intron, 5′-UTR, and intergenic) from the annotated 3′-UTR PACs in Ensembl genes was compared using the χ^2^-test (Wu *et al.* 2011). Furthermore, the 10-40 nts upstream region of the PACs was analyzed using the *SignalSleuth2* (parameters: k = 6 and once = T) to identify the potential over represented NUE motifs. Top five NUE motifs observed in PACs from each genomic region (3′-UTR, CDS etc) were used for the comparison among PACs from various genomic regions. The significance of the observed hexamers was measured as *Z-score* using the *RSAT* software (Nguyen *et al.* 2018).

### Gene Ontology (GO) analysis

Domain-based InterProScan GO terms annotations for the maize protein coding genes were obtained from the maize-*GAMER* (Wimalanathan *et al.* 2018). Singular Enrichment Analysis (SEA) tool (Du *et al.* 2010) with “*Plant GO slim*” parameter in the AgriGO web server (http://bioinfo.cau.edu.cn/agriGO/analysis.php) was used to find the association between GO terms and the specific gene sets. The significance of the GO enrichment was calculated using the *hypergeometric* test. The *Benjamini–Hochberg (FDR)* method was employed to adjust the *p-values* for the multiple hypothesis testing. GO terms with FDR *p-values* < 0.05 were considered significant. The keyword counts on the biological processes were used to summarize the GO terms associated with specific gene sets (Jeong and Nasir 2017).

### Comparison of PACs With known transcript ends

To compare PACs with annotated transcript termini, 131,496 known transcripts from 39,498 annotated protein-coding genes were obtained from the Ensembl (release-39). Of these, only 113,678 transcripts with an annotated 3′-UTR (regardless of 3′-UTR length) were selected. All non-protein coding genes were also excluded from further analysis due to lack of annotated 3′-UTRs. To simplify, only the 3′ most transcript terminus was taken from the genes with multiple transcripts. Furthermore, 136,745 PacBio transcripts were obtained from the previous study (Wang *et al.* 2018) and transcript termini that were within 24 nts of each other were iteratively clustered. Finally, 25,424 Ensembl transcript termini with 3′-UTR and 70,741 PacBio transcript termini were compared against the PACs generated in this study.

### Data availability

Publicly available datasets were analyzed in this study that can be obtained from the SRA database (https://www.ncbi.nlm.nih.gov/sra). The data generated and analyzed in this article is present within the article, figures, tables, and supplementary files. Supplemental material available at FigShare: https://doi.org/10.25387/g3.8034695.

## Results

### Identified cleavage sites in Maize using RNA-Seq data

To determine the poly(A) sites in the maize genome, a total of 401 samples from the 24 RNA-Seq datasets of the B73 maize variety were systematically retrieved from the SRA database (File S1 and S2). From the 9,485,003,995 total raw reads in the 24 datasets, 9,254,967,996 reads survived the low-quality and read length filtering criteria using the *Trimmomatic* software. The reads with eight or more A- or T-residues were considered as the polyadenylated reads and the rest were discarded. The 27,065,201 polyadenylated reads that remained unaligned to the maize genome in an initial alignment step using *Bowtie2* were retained further to ensure that the selected reads were post-transcriptional. Terminal A- or T-residues were removed from the unaligned reads and remapped to the maize genome using *Bowtie2*. Only 21,697,103 reads that mapped uniquely to the genome and were not internally primed were used to define cleavage sites in each sample. Summary of the total raw reads, number of filtered reads, total polyadenylated reads, uniquely mapped reads, and total number of cleavage sites obtained from each SRA dataset are listed in the Table S1. The cleavage sites from all 401 samples were pooled together to define 1,316,852 distinct cleavage sites in maize. Only 561,066 high quality cleavage sites that were supported by at least three mapped reads were retained for further analysis. All the cleavage sites that were within 24 nts of each other were iteratively clustered (referred as PAC from here onwards) to define 95,345 distinct PACs.

To assess whether the same numbers of PACs could be identified using fewer numbers of polyadenylated reads. The polyadenylated reads were randomly down sampled to define the number of PACs, starting from 20 million and decreasing one million with each step. The number of PACs decreased with decrease in the number of sampled reads suggesting that all (21 million) polyadenylated reads were required to identify 95,345 polyadenylation events (Table S2).

### Heterogeneity of cleavage sites

**Figure 1:**
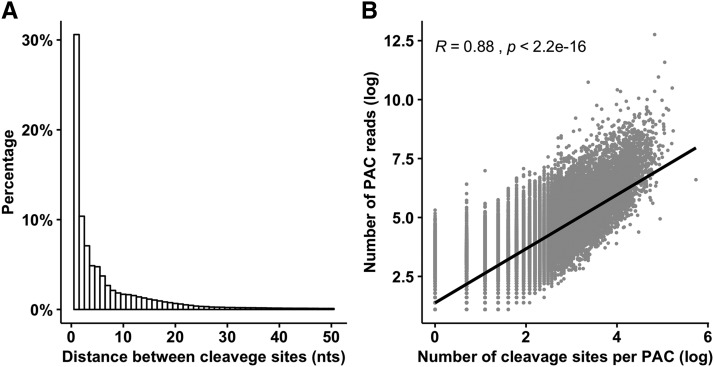
Micro-heterogeneity of cleavage sites in the maize genome. (A) Distribution of the distances between the adjacent cleavage sites. (B) Pearson correlation between *natural logs* of the number of cleavage sites and the number of supporting reads per PAC (*R* = 0.88, *p-value* = 2.2 × 10^−16^).

Heterogeneous nature of the 3′ end formation generates many cleavage sites that exist next to each other in the form of clusters (Pauws *et al.* 2001). A considerable heterogeneity was also observed in the maize cleavage sites and a large number of cleavage sites (83%) were within 24 nts of each other. The genomic distances between the adjacent cleavage sites were calculated and the relative frequency of the distances is shown in the Figure 1A. For the PACs containing multiple cleavage sites, the distance between the 5′-most cleavage site and 3′-most cleavage site was calculated. The distances for the majority of PACs (n = 68,453, 72%) were within 24 nts, highlighting that the choice of 24 nts window was sufficient to reduce the heterogeneity among the cleavage sites.

A total of 49,382 (52%) PACs had more than one cleavage sites with an average of 5.9 (SD = 11.5) cleavage sites per PAC. The cleavage process is stochastic and the discovery of heterogeneity depends mainly on the number of supporting reads (Tian *et al.* 2005). To study whether the number of cleavage sites per PAC in maize also increased with increase in the number of supporting reads, the Pearson correlation coefficient was measured between the natural logs of the number of supporting reads for the representative site in PAC and the number of cleavage sites per PAC (Figure 1B). The heterogeneous nature of the cleavage process was strongly correlated with the number of supporting reads (Pearson correlation = 0.88, *p-value* = 2.2 × 10^−16^).

### Genomic distribution of the PACs

**Figure 2:**
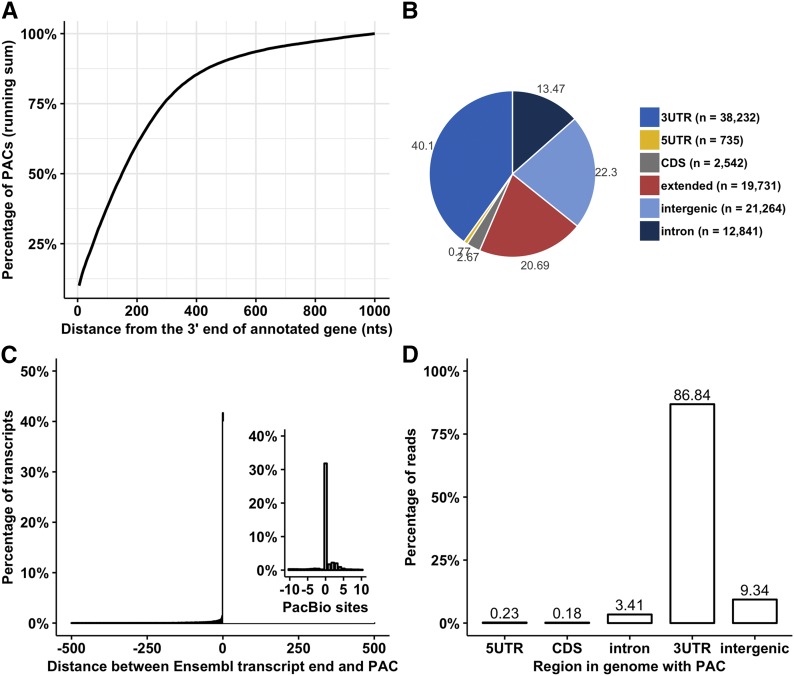
Assessment of genic and intergenic properties of the mapped polyadenylated reads. (A) The distribution of distances between the intergenic PACs and the 3′-end of the closest annotated genes. The relative frequency of PACs that were within the 1,000 nts of the annotated gene end was calculated in 5-nt intervals and a running sum of these values was plotted. (B) Distribution of PACs in genic and intergenic regions of the maize genome. (C) Most Ensembl transcript termini were within the boundaries of poly(A) clusters as shown in main graph. A similar trend was observed in case of PacBio transcripts (inset graph). (D) Distribution of polyadenylated reads mapped to different genomic features. As expected, most reads were mapped to the annotated 3′-UTR regions.

A total of 95,345 PACs were mapped to the Ensembl annotated transcripts in the maize genome (File S3). The maize genome consisted of a total 46,272 genes, of which 39,498 were protein-coding genes. The 46,272 genes expressed 138,270 transcripts, and of which 131,496 originated from the protein-coding genes. Only the longest transcript from each gene was selected to annotate the PACs if the genes had more than one annotated transcripts. About 40% of the PACs were distributed in the annotated 3′-UTR regions of the transcripts. The majority of the PACs (42.99%) were not located within any annotated gene (these PACs were termed as the intergenic PACs). Previous studies have shown that the majority of the intergenic PACs in plant species are located within few hundred nucleotides of the annotated genes (Wu *et al.* 2011; Zhao *et al.* 2014; Wu *et al.* 2015), highlighting the potential incompleteness of the 3′-UTR annotations in plants. About 37% (14,583 out of 39,498) of the longest annotated protein-coding transcripts in the maize genome also lacked an annotated 3′-UTR indicating that maize annotation is far from being complete as reported previously (Wang *et al.* 2016a, 2018). To further investigate the possible incompleteness of the 3′-UTR annotations in the maize genome, the intergenic PACs that were within 1,000 nts of the nearby-annotated 3′-end of the genes were analyzed (n = 24,594) (Figure 2A). Of these 24,594 intergenic PACs, approximately 50% of the PACs were situated within 150 nts of the annotated transcript termini. In maize, if indeed the intergenic PACs represent incomplete 3′-UTR annotations, intergenic PACs are expected to be more commonly associated with the genes that lacked annotated 3′-UTRs than the genes with annotated 3′-UTRs. To test this, PAC associated protein-coding genes were categorized into two groups. First group included 13,019 genes with an observed PAC in the 1,000 nts intergenic region, while the second group included 8,430 genes that had lacked a PAC in the 1,000 nts intergenic region. A total of 4,908 (38%) and 534 (6%) genes lacked an annotated 3′-UTR in group one and group two genes, respectively. This finding strongly suggested that the majority of the “intergenic” PACs could be probable real poly(A) sites that define the 3′ ends of the adjacent genes.

To portray the maize annotation more precisely, the 3′-ends of the transcripts with annotated 3′-UTR were extended by 150 nts based on the median of the intergenic PAC distribution in the Figure 2A. The 3′-ends of the transcripts that lacked annotated 3′-UTRs were extended by 460 nucleotides (where 310 was the median 3′-UTR length of all protein coding transcripts (n = 131,496) + 150 nts extension based on median of the intergenic distribution). By this definition, 20.69% (n = 19,731) of the PACs were classified as the “extended” 3′-UTR PACs that were associated with 12,361 genes (11,740 protein-coding genes). The mean distance between the extended PACs and the 3′-end of the closest upstream-annotated genes was 137 nts (SD = 119; median = 108). Figure 2B summarizes the distribution of the 95,345 PACs in different regions of the maize genome. A total of 77.70% PACs were located in the extended gene annotated regions, while remaining 22.30% were located in the intergenic region. The 60.79% of the total PACs were located within the extended 3′-UTR regions of the gene. The PACs in the introns, CDS and 5′-UTR regions were 13.47%, 2.67%, and 0.77%, respectively. All 95,345 detected along with updated assigned genomic regions are provided in File S3.

To evaluate how well identified PACs agree with the known transcript termini, PACs were compared with 70,741 and 25,424 PacBio transcript termini and Ensembl transcript termini, respectively (see Methods for details). Majority of the Ensembl and PacBio transcript termini showed an excellent agreement with the PAC cluster boundaries (Figure 2C). About 50.26% (n = 12,779) and 48.72% (n = 34,463) of the Ensembl and PacBio transcript termini were within 24 nts of the poly(A) clusters boundaries. A total of 36,866 PACs in our data were within 24 nts of either PacBio or Ensembl transcript termini. Of these, 10,376 were supported by both transcript sources. As expected, 98.30% and 85.48% of the supported Ensembl and PacBio transcript termini were either 3′-UTR or extended 3′-UTR PACs (Table S3). Among non-3′-UTR regions, supported PacBio sites were also observed for about 6%, 0.4%, 0.4%, and 8% PACs in intronic, CDS, 5′-UTR, and intergenic regions, respectively.

According to the Ensembl annotation, 22.30% (n = 21,264) PACs were located in the intergenic region. To assess whether these intergenic PACs overlap with the novel transcripts, the *de novo* transcripts were identified in the maize genome using 68 strand-specific RNA-Seq samples. About 47% (n = 9,975) intergenic PACs were validated by the *de novo* assembled genes (File S4).

To examine the contribution of different genomic region in the total uniquely mapped polyadenylated reads that were used to define 95,345 PACs, the percentage of the polyadenylated reads mapped to each genomic region was calculated. Most of the polyadenylated reads were mapped to the annotated genes in the maize genome. Of the genomic features, majority (86.84%) of the polyadenylated reads overlapped with the known extended 3′-UTR regions, demonstrating the agreement between the identified PACs and the known 3′-UTR gene annotations (Figure 2D). About 9% polyadenylated reads mapped in the intergenic regions, while only 0.18–3.41% of the polyadenylated reads were mapped to the non 3′-UTR regions of the genes.

### APA is widespread, however most APA genes express a predominant PAC

**Figure 3:**
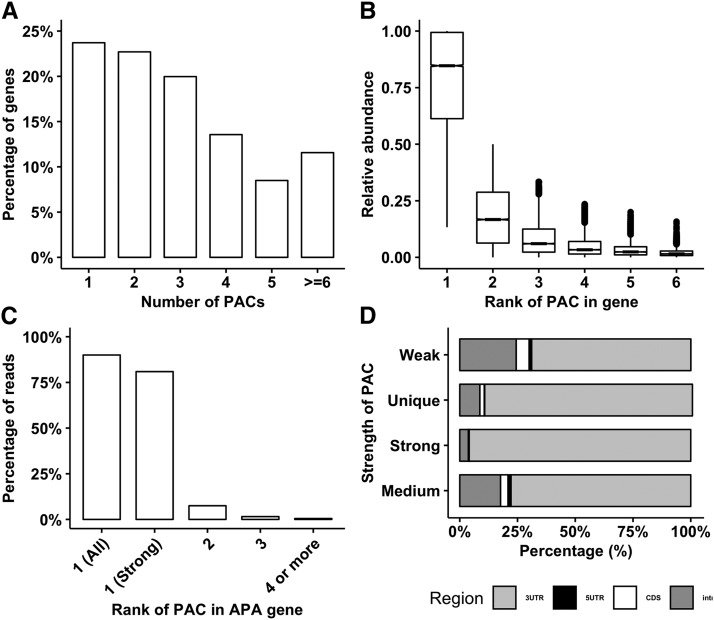
Analysis of alternative polyadenylation events. (A) Percentage of the number of PACs per gene. (B) Relative abundance of the PACs at each rank in the APA genes. For each APA gene, PACs were ranked based on the decreasing order of the relative abundances. In general, there exists one dominant PAC in APA genes. The difference between the first and second ranked PAC was statistically significant (wilcox.test *p-value* < 2.2 × 10^−16^, one-tailed greater) (C) Percentage of the total polyadenylated reads of the APA genes that were mapped to each position in the ranking. Approximately, 90% reads were mapped to the most abundant PAC. The “Strong” PACs from the 65% of the APA genes contributed the 80% of the total polyadenylated reads. (D) Majority of the non-3′UTR PACs either had a “weak” or “medium” expression levels.

Figure 3A summarizes the distribution of the number of PACs per gene in the maize *genome*. Overall, the 74,081 PACs were distributed in a total of 23,705 genes, which accounted for the 51% of the total annotated genes in the maize genome. A total of 23.71% (n = 5,620) of the gene contained a single PAC (termed as the constitutive genes). The two or more PACs were observed in 76.29% (n = 18,085) of the polyadenylated genes (termed as the APA genes), which corresponds to 68,461 (92%) of the total genic PACs. The APA genes with multiple PACs had an average of 3.79 (SD = 2.01 and median = 3) per gene. However, the quantification of multiple PACs within a gene based on RE indicated that in general most abundant PACs in the APA genes were expressed at much higher levels than the rest of the PACs, instead of expressing several PACs at a similar level (Figure 3B). The mean RE of the most abundant PAC (mean = 72%; median = 73%) was much higher than the mean RE for the second most abundant PAC (mean = 18%; median = 17%). The difference in the mean RE values of the most abundant and second most abundant PACs in the APA genes was statistically significant (wilcox.test *p-value* < 2.2 × 10^−16^, one-tailed greater).

In order to quantify the overall contribution of the most abundant PACs in the total polyadenylated reads mapped to the APA genes in maize genome, the percentage of reads mapped to the most abundant PACs out of total poly(A) reads was calculated (as described in the methods section). Approximately 90% of the poly(A) reads were mapped to the most abundant PACs of the APA genes, while of the remaining, 8% belonged to the second most abundant PACs (Figure 3C). To identify the potential predominant PACs within a given gene that were expressed at much higher levels than any other PACs in that gene, an empirical RE threshold of 0.70 was used. By this definition, a total of 13.48% (n = 9,985) PAC in 55.21% (n = 9,985) polyadenylated APA genes were considered as the strong PACs, while the remaining 32.82% (n = 24,313) PACs in these genes were termed as the weak PACs (Table 1). Around 44.78% of the APA genes (n = 8,100) with 34,163 (46.12%) PACs that lacked a predominant PAC were classified as the medium genes. The genes with strong PACs contributed majority of the total polyadenylated reads (Figure 3C). Most “non 3’-UTR” PACs were expressed at lower levels relative to 3′-UTR PACs that were either expressed at higher levels or were single PAC in a gene (Figure 3D). The complete list of enriched biological processes, molecular functions, and cellular components GO terms in APA, strong, and medium genes are provided in Table S4. The keyword count on the biological process gene ontology (GO) terms (Table S4) indicated that the APA genes were involved in the wide range of functions such as metabolic (n = 8 words) that includes both biosynthetic (4) and catabolic (1) processes, modification (2), regulation (2), localization (2), cell proliferation (1), generation of metabolites and energy (1), homeostasis (1), transport (1), response to stimulus (1), and signal transduction (1). Comparison of the enriched GO terms between the dominant and medium genes revealed that two sets of genes were involved in the different sets of functions. The keyword count on the biological processes for the strong genes revealed involvement in biosynthetic processes (n = 4 words), catabolic (1), energy and metabolites generation (1), translation (1), and signal transduction (1). The keyword count on the biological processes of GO terms for the medium genes indicated functional involvement in the regulation (n = 5 words), modification (2), transcription (1), development (1), and response to extracellular stimulus (1).

**Table 1:** Numbers of PACs in different PAC categories

PAC Classification	No. of PACs	PAC Percentage	No. of Genes	Gene Percentage
Unique	5,620	7.59	5,620	23.71
Strong	9,985	13.48	9,985	42.12
Weak	24,313	32.82	9,985	42.12
Medium	34,163	46.12	8,100	34.17
Total	74,081	100	23,705	100

### Polyadenylation in non-coding RNA genes

**Figure 4:**
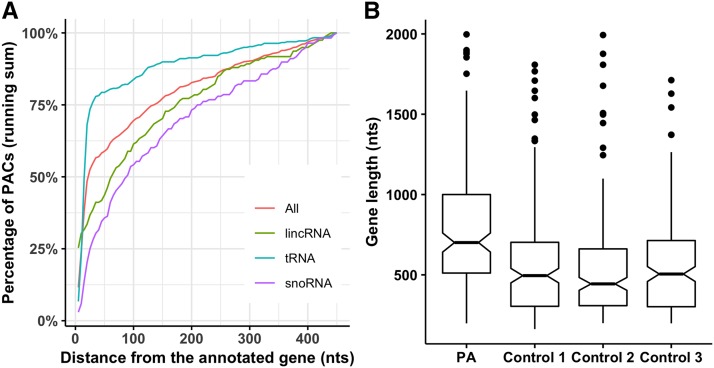
Analysis of polyadenylation events in non-coding RNA genes. (A) In addition to PACs in the intronic region of non-coding RNA genes, many PACs were located in the intergenic region within few bps of the annotated RNA genes. (B) Difference in lengths of LincRNA genes with and without PACs. LincRNA genes with PACs (PA) were significantly longer in length (wilcoxon.test *p-value* = 2.2 × 10^−16^) than those without annotated PACs (Control 1, Control 2, and Control 3).

More than 1,400 PACs were found in the non-protein coding genes (Table 2). All the PACs observed in the non-coding genes were either located in the extended 3′ ends (n = 788) or intronic regions of the genes (n = 646). Majority of the extended 3′ end PACs associated with RNA genes were within few base pairs of the annotated 3′ ends of the genes (Figure 4A). More than 50% of the extended PACs in all the RNA genes were located within 25 nts from the annotated 3′ end of the genes (Figure 4A). Among different classes of RNA genes, 50% of the extended PACs in the tRNA, lincRNA, and snoRNA genes were within 15 nts, 60 nts, and 85 nts of the annotated 3′ ends of the genes, respectively.

**Table 2:** Number of PACs in different non-coding RNA genes

Category*^a^*	Genomic Region		Total	Percentage (%)
	intron	Extended 3’-UTR		
lincRNA	470	158	628	43.79
tRNA	42	358	400	27.89
snoRNA	93	168	261	18.2
snRNA	17	65	82	5.72
SRP_RNA	18	10	28	1.95
Others	6	29	35	2.44
Total	646	788	1,434	100

a lincRNA: Long intergenic non-coding RNAs; tRNA: transfer RNA; snoRNA: small nucleolar RNA; snRNA: small nuclear RNA; SRP_RNA: signal recognition particle RNA; Others: other non-coding RNAs.

The highest numbers of PAC (n = 628) were observed in the lincRNA genes. These genes were analyzed further as lincRNA genes are known not to overlap with other genes (Ulitsky and Bartel 2013) and mapped PACs in this study can be assigned to these genes in an undisputed manner. Furthermore, only the intronic PACs in lincRNA genes were considered as the occurrence of PACs upstream of the 3′-most exons is much more common than the PACs in last exon of the lincRNA genes (Ziegler and Kretz 2017). A total of 470 intronic PACs were distributed in the 220 lincRNA genes of maize. Of which 115 were constitutive genes while remaining 105 were APA genes with more than one PACs. The APA genes on the average had 3.38 PACs per gene (SD = 1.97 and median = 3). To investigate the difference in gene lengths between lincRNA genes with and without PACs, gene lengths of all annotated lincRNA genes were extracted from the Ensembl gene annotations. As a negative control, three groups each of 220 genes were randomly selected (Control 1, Control 2, and Control 3) from the remaining 2,312 lincRNA genes in the maize genome with no observed intronic PAC. The mean lengths of the lincRNA genes with PAC and without PACs were 2,990 (median = 848) and 1,110 (median = 536), respectively (Figure 4B). The mean lengths of the polyadenylated lineRNA genes were significantly longer than the three pooled non-polyadenylated control datasets (wilcoxon.test *p-value* < 2.2 × 10^−16^).

### Intron and CDS with PACs were longer in length

**Figure 5:**
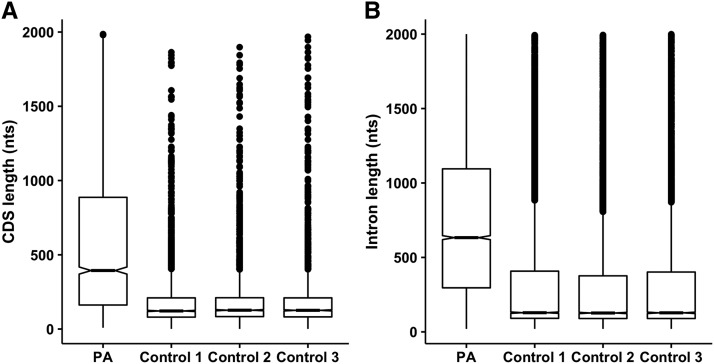
Difference in lengths of CDS and intronic regions with and without annotated PACs. CDS and introns with PACs (PA) were significantly longer in lengths than the lengths of those without annotated PACs (Control 1, Control 2, and Control 3). (A) CDS (one-tailed wilcoxon.test *p-value* = 2.2 × 10^−16^). (B) Introns (one-tailed wilcoxon.test *p-value* = 2.2 × 10^−16^).

A total of 2,542 PACs were detected in the CDS regions of the 1,809 protein-coding genes. To compare whether the CDS with PAC were significantly longer in length relative to the CDS without PACs (Wu *et al.* 2011; Zhao *et al.* 2014; Guo *et al.* 2016), three random samples each of 2,542 CDS without PACs were selected as the control datasets (Control 1, Control 2, Control 3). The mean lengths of the CDS with PACs and without PACs were 787 (median = 474) and 207 (median = 126), respectively (Figure 5A; one-tailed greater wilcox.test *p-value* < 2.2 × 10^−16^). Similarly, PACs in the introns showed preference for the longer introns. A total 12,195 intronic PACs were detected in a total of 6,193 genes. The mean length of the introns with PACs was compared against the three groups each of 6,193 randomly selected introns without any PAC (Figure 5B). The mean lengths of the introns with PAC and introns without PACs were 2,057 (median = 973) and 654 (median = 142) respectively (one-tailed greater wilcoxon.test *p-value* < 2.2 × 10^−16^).

### Conserved “U-A-U-A-U” single nucleotide pattern Around PACs

**Figure 6:**
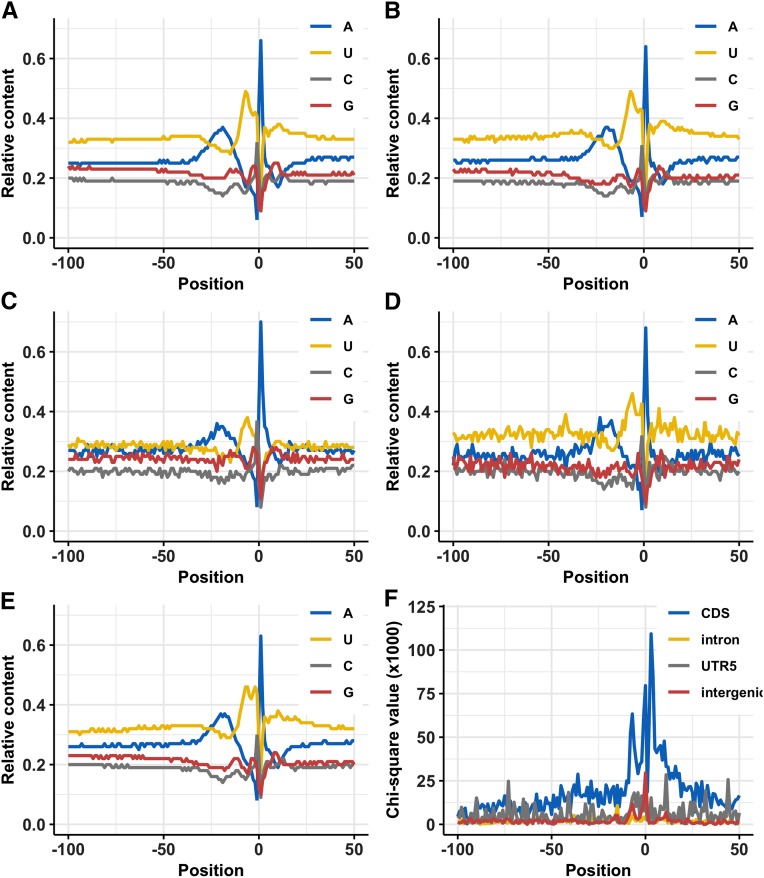
Average single nucleotide composition profiles computed in the surrounding -100 to + 50 base pairs around different PAC categories. The *x*-axis denotes position relative to the PAC location with negative numbers showing upstream region from the PAC and positive numbers showing downstream region from the PAC. The *y*-axis is the relative frequency of each base or chi-square values multiplied by 10^3^. (A) Nucleotide composition of PACs from the 3′-UTR region. (B) Nucleotide composition of PACs from the intronic region. (C) Nucleotide composition of PACs from the CDS region. (D) Nucleotide composition of PACs from the 5′-UTR region. (E) Nucleotide composition of PACs from the intergenic region. (F) In a pair-wise manner position-by-position nucleotide composition from the 3′-UTR PACs (“expected value”) was compared with the nucleotide composition from other regions (“observed value”) using the χ^2^ test. The χ^2^ values were multiplied with 10^3^ and plotted on *y*-axis.

To analyze the single nucleotide profiles, the sequences spanning -300 to +100 nt were extracted from the maize genome surrounding each PAC and profiles were generated using *SignalSleuth2* program. The analysis of longer upstream region (not shown) indicated a gradual transition from a relatively neutral nucleotide composition to a conserved “U-rich—A-rich—U-rich—A-site—U-rich” nucleotide composition. This conserved pattern coincides with the positions of the four groups of poly(A) signals found in plant kingdom including FUE, NUE, poly(A) site, and cleavage element before and after poly(A) site (Loke *et al.* 2005). Figure 6 depicts nucleotide composition in surrounding regions of different PAC types.

The single nucleotide profiles investigated in the 3′-UTR PACs (Figure 6A) indicated that the FUE region (-100 to -30 nts) was U rich (32–34%) and preferred nucleotides in the order U >> A > G >> C. The A content remained relatively stable (mean = 25%), while G and C content showed a gradual decrease from -100 to -30 positions. The NUE region (-28 to -13) had high A content with mean of 33% and peak at -19 (37%). The NUE region overall preferred nucleotides in the order of: A > U > G >> C, where U, G, and C content had an average of 30%, 21%, and 16%, respectively. The U-rich region -10 to -1 before poly(A) site coincided with the left cleavage element and showed the highest U content (mean = 43%) and peak at -7 (49%). This region preferred nucleotides in the order of: U >> G > C > A, where rise in U content could be attributed to the sudden drop in the previously stable or high A content (mean = 17%). At -1 position, overall the preferred nucleotide order was U >> C >> G >> A. The A content dropped to 6% at this position. At poly(A) site (0 position), A was the preferred residue followed by the U, C, and G, respectively. The U-rich region (+1 to +10) that coincides with the right cleavage element allowed nucleotides in the order of: U >> A >> G > C. This region can be differentiated from the upper U-rich region (-10 to -1) in terms of lower U content (mean: 34% *vs.* 43%) and higher A content (mean: 28% *vs.* 17%). The C and G contents showed almost similar occurrences in both left and right cleavage element regions.

More than 16,000 (16.91%) of the PACs were located in the annotated introns, CDS, or 5′-UTR regions of the genes. Nucleotide compositions around these “non-3’-UTR” and intergenic PACs are shown in Figures 6B-E. The single nucleotide compositions from these PACs were compared in position-by-position manner with single nucleotide composition of PACs from the 3′-UTR regions using the χ^2^ test (Figure 6F). As expected, the intronic PACs were indistinguishable from the 3′-UTR PACs. The single nucleotide profiles of intergenic PACs were similar to profiles noted in the 3′-UTR PAC except at and around poly(A) site where U content was higher. The χ^2^ values indicated that the PACs in the 3′-UTR and CDS were very different. The variation can be attributed to the overall decrease in the U content and rise in A+G+C content in the CDS PACs (Figure 6A and Figure 6C). Moving toward the poly(A) site, the two PAC groups diverged gradually in the FUE region (-100 to -30) with lower U content in CDS (29%) than the 3′-UTR (33%), however a typical 3′-UTR like conserved pattern (U > A > G > C) was still observed. The NUE region (-28 to -13) in CDS had a similar high A content (mean = 32%) with a peak further three bases upstream than the 3′-UTR at -22 position (36%). The region indicated lower U content and higher G+C content in the CDS PACs. Although, the cleavage element left (-10 to -1) in CDS preferred the same nucleotide order as 3′-UTR (U >> G > C > A), the overall U content was much lower than the 3′-UTR (34% *vs.* 43%). The cleavage element right (+1 to +10) of CDS PACs preferred A over T unlike 3′-UTR PACs. The preferred nucleotide order in the CDS PACs was A >> U > G > C. At -1 position, a clear preference for the C nucleotide was observed in CDS PACs. Similar to the 3′-UTR PACs, the poly(A) site (0) showed preference for the A residue, however among other nucleotides C was more preferred than U.

### Comparison of NUE motif frequency in different PAC types

Previous studies indicated that the plant poly(A) sites are surrounded by various poly(A) signals (Loke *et al.* 2005). Among these, the NUE is considered as the most conserved poly(A) signal. To compare PACs from the different genomic regions, the poly(A) signals in the -40 to -10 NUE region of different genomic PAC were examined using the *SignalSleuth2* and *RSAT* programs. The overall frequency of occurrence and the significance (measured in terms of *Z-score*) of the observed hexamers were compared (Table S5). The frequency distributions of the hexamers revealed that the top five motifs were similar in the NUE region of 3′-UTR, introns, and intergenic region with few motifs changing their ranks. However, in the intergenic region CG rich motifs were more significant among the top motifs. Canonical AAUAAA was the most frequent and significant motif in the NUE region of 3′-UTR, introns, and intergenic PACs. However, it was observed in only 5% of the PACs in all three PAC types. In CDS PACs, the AAUAAA was not among the top frequent motifs and frequency was low (2.71%) compared with other regions. The NUE region in CDS indicated higher occurrence of G + A rich motifs relative to U + A rich motifs found in 3′-UTR, intronic, and intergenic PACs. The motif composition was in accordance with a drop in U content and rise in G content observed in the single nucleotide profiles of the CDS PACs. Only two motifs were detected in the 5′-UTR PACs and among these AAUAAA was not detected. The 5′-UTR motifs were different from those observed in the other regions, however, this could be attributed to the low number of PACs in 5′-UTR.

About 3,016 PACs were associated with the canonical AAUAAA motif in the upstream NUE region. These PACs were distributed either in 3UTR (1,882) or extended 3′-UTR (1,134) regions of the 2,830 maize protein-coding genes (File S5). Out of 2,830 genes, the 454 genes were constitutive (*i.e.*, had single PAC), while rest of the genes were APA genes. To further examine which specific sets of genes that still maintain the use of AAUAAA as the poly(A) signal in maize, the enriched GO terms in genes containing AAUAAA were investigated. The analysis indicated that such genes are functionally involved in the metabolic biological processes including cellular amino acid and derivative metabolic process (GO:0006519, number of genes n = 86, FDR = 1.5 × 10^−08^), carbohydrate metabolic process (GO:0005975, n = 133, FDR = 5 × 10^−6^), catabolic process (GO:0009056, n = 98, FDR = 8.4 × 10^−5^), secondary metabolic process (GO:0019748, n = 16, FDR = 7.1 × 10^−3^), and generation of precursor metabolites and energy (GO:0006091, n = 37, FDR = 1.6 × 10^−2^). Furthermore, evaluation of the relationship between the occurrence of AAUAAA and expression level of PACs in APA genes (n = 2562 PACs) indicated that AAUAAA motifs were more strongly associated with PACs showing strong (32.40%, n = 830) or medium (45.32%, n = 1161) expression relative to the weak expression (22.29%, n = 571).

## Discussion

Availability of large numbers of transcriptomic sequences provides an unprecedented opportunity to explore genome-wide polyadenylation profiles. The standard RNA-seq reads are commonly used to define the genome wide polyadenylation profiles in species with no available or limited polyadenylation data (Zhao *et al.* 2014; Wang *et al.* 2016b). Here, 9.48 billion RNA-Seq reads from the 24 high-throughput transcriptomic studies were analyzed to comprehensively characterize genome-wide polyadenylation profiles in the maize. The datasets used in this study consists of 401 pooled samples collected from different tissues under various physiological and developmental conditions (Table S1). Using a robust PAC identification process (Dong *et al.* 2015) with slight modifications, 21 million transcriptomic reads with eight or more terminal A- or T-residues were used to find the evidence for 95,345 polyadenylation events (PACs) in the maize genome.

Heterogeneity in the polyadenylation events is a common phenomenon that indicates the imprecise nature of the cleavage process in general (Tian *et al.* 2005; You *et al.* 2014). In this phenomenon, cleavage occurs at multiple locations that are few bases apart (You *et al.* 2014). This study demonstrated that 52% of the PACs in maize have more than one cleavage sites, which is similar to the proportion of heterogeneity observed in *human* (51%) and *mouse* (47%) (Tian *et al.* 2005). Similarly, heterogeneity was also noted in the previous studies on plants including *Arabidopsis* (Wu *et al.* 2011), *rice* (Shen *et al.* 2008), *Mediga truncatula* (Wu *et al.* 2014), *chlamadomoas reinhardtii* (Zhao *et al.* 2014). A strong positive correlation (0.88) observed between the numbers of supporting reads and the number of cleavage sites per PAC supports the stochastic nature of the cleavage process, as reported previously (Tian *et al.* 2005). The heterogeneous cleavage sites in maize may provide valuable insights about the mechanisms of cleavage sites selection and can be compared to heterogeneity datasets reported in other species (You *et al.* 2014).

Ensembl gene annotations (version 39) with more than 46,000 (∼39,000 protein coding) genes were used to annotate PACs. However, about 37% of the protein coding transcripts lacked an annotated 3′-UTR making annotation of PACs more challenging. According to Ensembl annotations, majority (43%) of the identified PACs are distributed in the intergenic regions. In general we observed that the intergenic PACs close to the 3′-ends genes are more commonly associated with transcripts that lacked an annotated 3′-UTR (38% *vs.* 6%) indicating that the existing 3′-UTR annotations are probably incomplete or inaccurate. The occurrence of PACs in intergenic regions could be attributed to incomplete 3′-UTR (Lopez *et al.* 2006), novel unannotated transcripts (*Hanada et al.* 2007) or orphan genes (Schmitz and Bornberg-Bauer 2017). Similar reports of incomplete 3′-UTR annotations were also reported in related plants (Wu *et al.* 2011; Zhao *et al.* 2014). After expanding the 3′-ends of transcripts, majority (78%) of the PACs are observed within the genic region, while remaining PACs are distributed in the intergenic PACs. Of these intergenic PACs 47% were present within the novel *de novo* transcripts. Similarly, intergenic PACs in Arabidopsis were also found to be associated with the *de novo* transcripts (Wu *et al.* 2015). However, around 53% PACs in maize are still localized in the intergenic regions and the significance of these intergenic sites in identifying novel transcripts in maize needs further verification in the future studies. Consistent with the previous studies (Wu *et al.* 2011; Ziegler and Kretz 2017), most poly(A) sites are found in the 3′-UTR regions, whereas only small fraction of PACs are situated in the other non-3′-UTR regions. The introns and CDS with PACs are longer in length than without PACs and this is similar to results reported in mammals and plants (Tian *et al.* 2007; Wu *et al.* 2011; Zhao *et al.* 2014; Guo *et al.* 2016). The preference of PACs in longer introns has been linked with the interaction between splicing and polyadenylation (Tian *et al.* 2007). Also, such PACs are known to occur more often in terminal introns (Wu *et al.* 2014; Movassat *et al.* 2016;). A comprehensive study is required to further uncover the significance and biasness of PACs toward the longer introns and CDS across various eukaryotic species.

At least one PAC is reported for the 51% of the total maize genes annotated in Ensembl version 39. About 23% of these genes are constitutive and use single predominant PAC site. However, more than 76% these genes use multiple PACs indicating that the APA is a widespread mechanism in maize, which is similar to 50–70% APA events reported in other plants (Shen *et al.* 2008; Wu *et al.* 2011; Zhao *et al.* 2014; Fu *et al.* 2016). To understand how APA is regulated, we evaluated the relative expression of PACs within APA genes. In APA genes, not all PACs are expressed at equal levels (Figure 3B). Dominant PACs are expressed at much higher levels than rest of the PACs in the genes and contributed 90% of the polyadenylated reads. Most APA PACs are expressed at low levels and may be attributed to transcriptional noise. Similar trend of APA regulation was observed in mammals (Derti *et al.* 2012). Further investigation of APA strengths in a separate study indicated that most APA events except the most dominant are deleterious for the cell (Xu and Zhang 2018). Furthermore, most protein-coding genes that expressed multiple transcripts in humans also preferred a single dominant transcript and transcription start site rather than expressing multiple isoforms at equal levels (Gonzàlez-Porta *et al.* 2013). Using a gene-centric approach, we quantified the number of genes that expressed strong PAC relative to others. More than 55% of APA genes expressed a strong PAC and all other PACs in such genes are expressed at much lower levels. And, even still a significant portion of APA genes (44%) expressed more than one preferred PACs (termed as medium) in our analysis.

Fewer PACs resides in the non-coding RNA genes. According to the current genome annotation, these PACs are situated in the extended 3′ ends or introns of these genes. In different classes of non-coding RNA genes, 50% of the extended PACs are within few bases of the annotated gene ends (Figure 4A). However, the exact nature of these 3′ end extensions remains unresolved as 3′ end extension criterion used in this study is mainly dominated by the PACs originating from the protein-coding genes (see results section). Furthermore, poly(A) tails are known to play important role in the degradation of defective tRNA, rRNA, and snRNA genes (Li and Du 2013). Considering these factors, only lincRNA genes with at least one annotated intronic PAC were evaluated further, as these are known to posses mRNA like features including poly(A) tail and these do not overlap with other genes (Ulitsky and Bartel 2013). Analysis of 470 intronic PACs in the lincRNA genes indicates that 48% of the 220 genes are APA genes, whereas rests are constitutive genes. Similarly, the APA events in the introns of human and mouse lncRNA genes were also reported previously (Ziegler and Kretz 2017). Previous studies have revealed that polyadenylated lncRNAs genes are longer in length than non-polyadenylated lncRNA genes (Wang *et al.* 2014; Liu *et al.* 2015). Consistent with the previous studies, in maize the lincRNA genes with PACs are significantly longer in length than the lincRNA genes without any PACs (Figure 4B).

Conservation of nucleotide composition surrounding the polyadenylation sites acquired from the NCBI mRNA sequences has been studied across animals, plants, and microorganisms (Li and Du 2013, 2014). This nucleotide composition pattern is generally correlated with the location of key *cis*-elements around PACs (Loke *et al.* 2005). Nucleotide composition in maize is similar to the nucleotide composition reported previously in maize and closely related plant species (Loke *et al.* 2005; Li and Du 2014; Fu *et al.* 2016). The analysis of longer upstream region clearly indicated a gradual transition from a relatively neutral nucleotide composition to a conserved “U-rich—A-rich—U-rich—A-site—U-rich” nucleotide composition reported in plants (Li and Du 2014). This conserved pattern corresponds to “FUE—NUE—CE—Cleavage site—CE” order of *cis*-element (Loke *et al.* 2005). The nucleotide composition of 3′-UTR was in agreement with nucleotide composition of PACs in the intronic, intergenic, and 5′-UTR regions. Single nucleotide profiles around these PACs are overall A + T rich. However, CDS profiles are more G-rich and different from the nucleotide composition noted in other PAC types. These nucleotide composition variations are similar to results reported in Arabidopsis and Green Algae (Wu *et al.* 2011; Zhao *et al.* 2014). At cleavage site, a peak of A residues is observed which is consistent with reports in plants (Li and Du 2013, 2014). At -1 position, U was preferred over C as reported (Li and Du 2013). In contrast, *Li and Du* observed that the C is more enriched than U, however, the difference in the relative frequencies of U and C residues was minor (Li and Du 2014). In this study, preference of C was observed at this position only in case of CDS PACs.

Previous investigations of motifs in the NUE region of maize based on NCBI mRNAs and transcript ends assembled from the RNA-Seq data indicated that the canonical hexamer AAUAAA has the highest frequency (Li and Du 2014; Wang *et al.* 2018). The top three observed NUE motifs (AAUAAA, AUAUAU, UAUAUA) in this study are consistent with previous reports (Wang *et al.* 2018). The AAUAAA is the top most frequent and significant occurring motif in 3′-UTRs, introns, and intergenic regions. The CDS and 5′-UTR preferred different NUE motifs over AAUAAA. The different nucleotide profiles and NUE motifs for the CDS region relative to the 3′-UTR region were also reported in case of green algae (Zhao *et al.* 2014). However, the estimates of NUE motif distribution in the 5′-UTR regions needs further validation in future upon availability of more data.

The frequency of AAUAAA observed in 3′-UTR is lower than reported previously in maize (Wang *et al.* 2018). Two factors might have resulted in overestimation of the AAUAAA motif occurrence in the previous studies (Li and Du 2014; Wang *et al.* 2018). First, use of un-clustered transcript ends to estimate the NUE motif frequency relative to the 24 nts clustered PACs used in the current study. Second, a much wider search region (-50 to -1 and -40 to -1) used in previous studies relative to -40 to -10 nts region used in this study. According to our data, about 2,800 genes still maintained the use of AAUAAA predominantly in the upstream regions of the strong or medium PACs (File S5). These genes with AAUAAA motif are involved in core biological processes including cellular amino acid and derivative metabolic process, carbohydrate metabolic process, catabolic process, secondary metabolic process, and generation of precursor metabolites and energy. Further research is required to understand the functional and evolutionary importance of genes that still use AAUAAA motif.

Although large numbers of the PACs were identified using RNA-Seq data, however limited poly(A) reads (0.29%) were detected in whole dataset, which is typical of standard RNA-Seq reads (Wang *et al.* 2016b). Different number of poly(A) reads were identified in each SRA dataset. However, for the present study only few SRA datasets provided majority of the poly(A) reads. Highest numbers of reads were observed in samples associated with early development followed by the germinating seedlings. This could be due to the fast growing nature of these tissues leading to tissue-specific APA regulation. Significantly higher rate of distinct APA events were also observed previously in the seedlings of Arabidopsis (Shen *et al.* 2011). Considering the over representation of certain libraries in our data, it is likely that the difference in the abundance of different PACs (based on RE values) for the same gene might simply reflect the difference in the proportion of polyadenylated reads from different tissue/cell types. However, even with low coverage in other tissues, highly expressed poly(A) events have more chances of being observed relative to ones with low expression.

The polyadenylation events reported here are still underrepresented and cover only 51% of the maize genes due to low coverage of poly(A) tails in the RNA-Seq data. Its challenging to comprehensively identify all polyadenylation events in each tissues using RNA-Seq. Alternatively, to increase coverage MPSS (Meyers *et al.* 2004) high throughput technology can be employed to profile poly(A) sites in maize. Moreover, PAT-seq had been used extensively to profile poly(A) sites in model plant species (Wu *et al.* 2011, 2014; Harrison *et al.* 2015;). Many other poly(A) specific protocols including PolyA_seq (Derti *et al.* 2012), and 3′ READS+ (Zheng *et al.* 2016) could also be considered in order to overcome the limited number of poly(A) reads observed in this study. Furthermore, there is an immense need of studying correlation of maize poly(A) data with additional data types such as transposable elements (Lee *et al.* 2008) and single nucleotide polymorphism (Xu and Zhang 2018). The study, without a doubt, will help further research on the APA patterns in maize. The findings of this study will improve our understanding about the gene expression regulation in maize.
